# A white man with Kikuchi-Fujimoto disease mimicking lymphoma, preceded by frequent episodes of tonsillitis: a case report

**DOI:** 10.1186/s13256-017-1208-4

**Published:** 2017-02-12

**Authors:** Agata Szczurowska, Tomasz Pawlowski, Agnieszka Halon, Anna Skoczynska

**Affiliations:** 1 Department of General Radiology, Interventional Radiology and Neuroradiology, Wroclaw Medical University, University Hospital in Wroclaw, Borowska 213 50-556, Wroclaw, Poland; 2 Department and Clinic of Internal and Occupational Diseases and Hypertension, Wroclaw Medical University, University Hospital in Wroclaw, Borowska 213 50-556, Wroclaw, Poland; 3 Division of Pathomorphology and Clinical Cytology, Department of Pathomorphology, Wroclaw Medical University, University Hospital in Wroclaw, Borowska 213 50-556, Wroclaw, Poland

**Keywords:** Kikuchi-Fujimoto disease, Tonsillitis, Cervical lymphadenopathy

## Abstract

**Background:**

Kikuchi-Fujimoto disease is a very rare cause of benign lymphadenopathy affecting mainly young Asiatic females. Little is known about the causative agent of Kikuchi-Fujimoto disease; however, there are hypotheses of infectious, autoimmune, or hyperimmune background of the disease that have not yet been confirmed in the conducted studies. Frequent episodes of tonsillitis preceding the onset of Kikuchi-Fujimoto disease have not been described as yet.

**Case presentation:**

A 23-year-old white man with a history of over 20 episodes of tonsillitis in the preceding 2 years was admitted to our hospital because of cervical unilateral lymphadenopathy, fever, night sweating, weight loss, and fatigue. On admission, slight tenderness of cervical lymph nodes and asymmetric palate tonsil enlargement were noted. Owing to the patient’s general symptoms and history of malignancy in his close family, a malignant disease such as lymphoma was suspected. Histopathological examination of the excised lymph node revealed areas of coagulative necrosis with abundant karyorrhectic debris, with histiocytes and lymphocytes observed at the margins of the necrotic areas. The microscopic examination led to an unexpected diagnosis of Kikuchi-Fujimoto disease. The patient was treated symptomatically. All of the patient’s symptoms, excluding tonsil enlargement, retreated within 2 months.

**Conclusions:**

In the differential diagnosis of cervical lymphadenopathy in patients with frequent episodes of tonsillitis, Kikuchi-Fujimoto disease should be taken into account. Kikuchi-Fujimoto disease may convincingly mimic symptoms characteristic of lymphoma.

## Background

Kikuchi-Fujimoto disease (KFD), or histiocytic necrotizing lymphadenitis, is an extremely rare entity belonging to the large group of diseases that may cause lymph node enlargement. KFD occurs mainly in young Asian females [1]. Case reports concerning white male patients are scarce. In Poland, only 11 cases of KFD have been described so far [1]; therefore, knowledge of this disease among physicians is minimal. Symptoms of KFD are not specific and include lymphadenopathy (usually cervical) with tenderness, fever, and night sweating, less frequently accompanied by sore throat, arthralgia, gastrointestinal symptoms, or skin involvement [2, 3]. A clinical presentation of KFD may mimic both more common and more severe diseases, including infections and lymphomas. Histopathological examination of a lymph node is an approved diagnostic measure for confirming KFD. Typical findings include the presence of coagulative necrotic areas with groups of several types of histiocytes at their margins and the absence of granulocytic infiltration [2]. The cause of KFD has not been established yet. Theories about *Yersinia enterocolitica* and *Toxoplasma gondii* being the causative agents have not been supported by studies conducted so far [4]. There are suspicions that viral infections, such as with human herpesvirus (HHV)-6, HHV-8, Epstein-Barr virus (EBV), parvovirus B19 or human T-cell lymphotropic virus 1, presumably combined with individual predispositions, may result in the development of KFD. The hypotheses on the possible causative agents lack supportive data [2, 5]. Another theory postulates an autoimmune background for KFD in association with systemic lupus erythematosus and antinuclear antibody (ANA) positivity in some patients [3]. KFD may also possibly develop as a result of a hyperimmune reaction to various stimuli, including different types of microorganisms, in susceptible individuals [2]. However, to our knowledge, there has not yet been a case report of KFD in a patient with recurrent tonsillitis.

## Case presentation

A 23-year-old white man with a history of over 20 episodes of bacterial tonsillitis in the preceding 2 years was admitted to our hospital with enlarged left cervical lymph nodes. The lymphadenopathy had appeared suddenly 2 weeks prior to admission and had not retreated despite the application of several types of antibiotics. One week prior to admission, the patient had started to manifest the following symptoms: fever reaching 39 °C, fatigue, weight loss, and intense night sweating. In the anamnesis on admission, the patient reported that his brother had died at a young age owing to a malignant tumor located in the nasopharyngeal region. He also admitted that he had been working in a muddy area and had been scratched by a pet rat several months earlier. He denied having traveled to regions of endemic diseases. His physical examination on admission revealed a few separate enlarged lymph nodes on the left side of his neck. The lymph nodes were slightly tender, and the skin covering them was unchanged. His palate tonsils were asymmetrically enlarged (in favor of the left side) without any discharge. He had a fever that reached as high as 39.5 °C and was successfully lowered only by metamizole administered parenterally. No other significant abnormalities were found.

A possible differential diagnosis was formed on the basis of the patient’s anamnesis and clinical examination. Additional diagnostic tests for infectious diseases and malignancies, including Hodgkin or non-Hodgkin lymphoma as well as carcinoma of the nasopharynx or the tonsils, as possible causes of cervical lymphadenopathy were performed.

Leukopenia (2.2–3.32 × 10^3^ cells/μl), moderate anemia (hemoglobin 12.0 g/dl), and thrombocytopenia (127 × 10^3^ cells/μl) were observed. A blood smear test revealed granulocytopenia, monocytosis, and 1% of lymphoid cells. A slightly elongated activated partial thromboplastin time (39.8 seconds) and increased D-dimer level (1.9 μg/ml) without other coagulation abnormalities were revealed. Inflammatory markers were increased, with the patient’s C-reactive protein (CRP) level reaching 17.63 mg/L and erythrocyte sedimentation rate amounting to 40 mm/hour, accompanied by negative procalcitonin. The patient’s serum lactate dehydrogenase (LDH) level was also increased at 463 U/L. During hospitalization, a threefold increase in CRP and nearly a doubling of LDH level were observed (Table 1).

**Table 1:** Abnormal results of laboratory tests and normal ranges of measured parameters

Parameter	Values	Reference values
aPTT, seconds	39.8	25–37
D-dimer, μg/ml	1.9	0–0.5
CRP, mg/L	17.6–62.3	0–5
ESR, mm/hour	40	1–10
LDH, U/L	413–762	0–248
WBC, 10^3^ cells/μl	2.2–3.32	4–10
PLT, 10^3^ cells/μl	127	150–450
Hemoglobin, g/dl	12.0	14–18

*Abbreviations: aPTT* Activated partial thromboplastin time, *CRP* C-reactive protein, *ESR* Erythrocyte sedimentation rate, *LDH* Lactate dehydrogenase, *WBC* White blood cell count, *PLT* Platelet count

Reference values of measured parameters are presented in accordance with the norms applied at the laboratory of the University Hospital in Wroclaw, Poland. All presented parameters were determined in the blood

Serological tests detecting toxoplasmosis (immunoglobulin G [IgG], IgM), Venereal Disease Research Laboratory test, hepatitis B surface antigen (HBs), anti-HB core antigen antibodies, anti-hepatitis C virus antibodies, EBV antibody to viral capsid antigen (IgG, IgM), anti-human immunodeficiency virus (HIV) antibodies, anti-streptolysin O antibodies, and ANA were negative. Anti-HBs antibodies were present at a concentration of 36.94 mIU/ml (as a result of vaccination), whereas anti-cytomegalovirus antibodies were IgG-positive and IgM-negative. The results of bacteriological cultures of blood and urine, as well as the tuberculin skin test, were negative. A chest x-ray revealed no abnormalities. Craniofacial computed tomography (CT) with contrast administration revealed numerous cervical lymph nodes enlarged up to 2 cm in diameter on the left side, with the majority of them situated in the supraclavicular area and a few of them located under the sternocleidomastoid muscle as well as in the submandibular region and next to the mandibular angle. Several slightly enlarged lymph nodes were also found on the right side under the mandible. CT of the chest revealed several lymph nodes up to 0.8 cm in width in the left axillary region. The lungs and mediastinum were not altered.

**Fig. 1:**
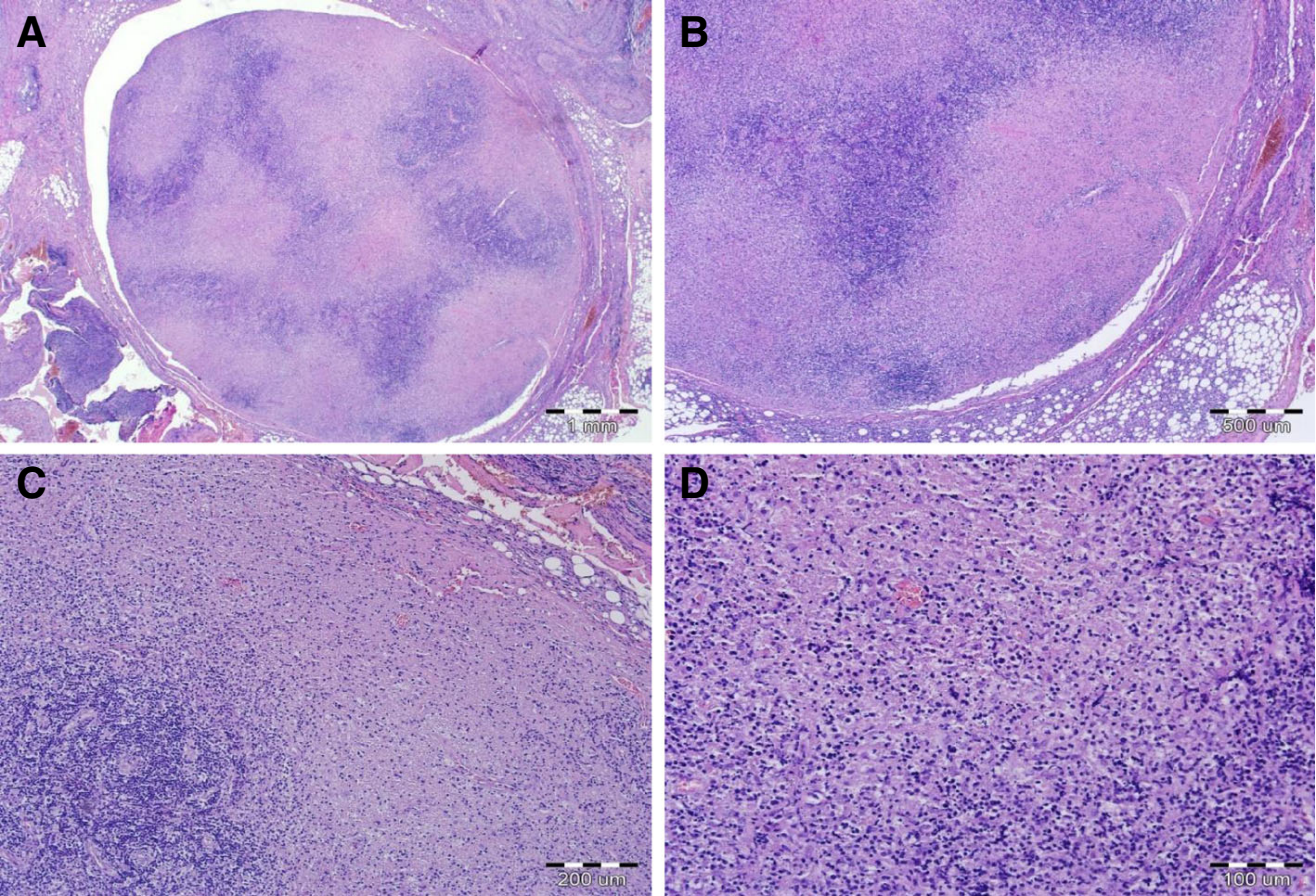
Histopathological findings of the lymph nodes. **a** Irregular paracortical areas of coagulative necrosis with abundant karyorrhectic debris and distortion of the nodal architecture (hematoxylin and eosin stain, original magnification ×20; scale bar = 1mm). **b** Areas of paracortical hyperplasia with small and large lymphoid cells (hematoxylin and eosin stain, original magnification ×40; scale bar = 200 μm). **c** Extensive paracortical area of coagulative necrosis with different types of histiocytes and lymphocytes at the margins of the necrotic areas (hematoxylin and eosin stain, original magnification ×100; scale bar = 200 μm). **d** The karyorrhectic foci formed by different cellular types, predominantly histiocytes, plasmacytoid monocytes, and small and larger lymphocytes (hematoxylin and eosin stain, original magnification ×200; scale bar = 100 μm)

The patient underwent fine-needle aspiration cytology (FNAC), with a normal result. Subsequently, an excision of the enlarged lymph nodes was performed. A histopathological examination of the lymph nodes with immunohistochemistry produced an unexpected result. In all three excised lymph nodes, an identical morphological image was observed: irregular paracortical areas of coagulative necrosis with abundant karyorrhectic debris and distortion of the nodal architecture. Different types of histiocytes and lymphocytes were observed at the margins of the necrotic areas. The karyorrhectic foci were formed by different cellular types. Areas of paracortical hyperplasia with small and large lymphoid cells were observed (Fig. 1).

Cluster of differentiation 8 (CD8) and CD4 antigens were positive in the lymphocytes located in the clusters. CD138 was positive in plasmocytes, CD15 was positive in a few cells, and CD30 was negative. On the basis of the histopathological findings, a diagnosis of KFD was made. Afterward, the patient remained in the hospital for a several-days-long observation period, during which symptomatic treatment was continued and fever as well as sweats retreated. In the 3-month follow-up after the patient’s discharge from the hospital, he was found to be in a good general state with no sign of lymphadenopathy. In a 6-month-long observation period, the only abnormality was the asymmetry of the palate tonsils. The patient was qualified for elective tonsillectomy.

## Discussion

KFD is a self-limiting disease; the symptoms retreat spontaneously within 6 months. There is no causal treatment of KFD; the disease is treated symptomatically with antipyretic and anti-inflammatory drugs, or with steroids and hydroxychloroquine in more severe cases [5, 6]. The prognosis is very good, and there is a low probability of a recurrence.

In our patient, the clinical symptoms and initial period of the disease were typical according to the literature [2, 4, 7]. However, the symptoms, including lymphadenopathy and B-type-like symptoms, mimicked features that occur in the course of lymphomas. At first, different types of lymphoma were taken into consideration. Owing to the patient’s epidemiological features, including age and sex [8], Hodgkin lymphoma was suspected at first. Aggressive subtypes of non-Hodgkin lymphoma were also taken into account in the differential diagnosis. The only symptom that was not consistent with a suspicion of lymphoma was the transient tenderness of the enlarged lymph nodes. Carcinoma of the nasopharynx or the palate tonsil were also considered, although laryngological examination and CT did not reveal lesions typical for a carcinoma. The differential diagnosis in KFD is based on the main causes of lymphadenopathy and, besides different types of lymphoma, includes infections with *Bartonella henselae* (a so-called cat-scratch disease), EBV, HIV, toxoplasmosis, and tuberculosis [5], as well as metastases.

Besides a neoplasm, we also suspected a viral infection such as mononucleosis or mumps in our patient. However, viral infections were excluded on the basis of our patient’s lack of contact with patients with infectious diseases; the absence of symptoms such as cough, skin lesions, or hepatosplenomegaly; and negative results of specific serological tests. The diagnosis of a bacterial infection also seemed improbable because of the lack of antibiotic therapy efficacy, negative bacteriological cultures, and negative tuberculin skin test results.

KFD occurs mainly in young females, especially of Asian origin. There are some cases of the disease described in non-Asian countries. Dumas *et al*. presented a study of 91 patients with KFD in France between 1989 and 2011, in which 76.9% of the studied population was female [7]. In our case, the patient’s ethnicity and sex were not typical of KFD.

Owing to the lack of evidence for the causes of KFD in the literature, the probable background for the disease in our patient remains unknown. However, attention should be drawn to the numerous episodes of tonsillitis in the patient’s past. To our knowledge, this factor has not been discussed in the literature. Nevertheless, unspecific frequent upper respiratory tract infections preceding KFD development have been mentioned [9]. Recurrent tonsillitis can be caused by chronic staphylococcal infection [10]. Assadian *et al*. found lymphocytes with human adenovirus and/or EBV in all surgically removed tonsils of their patients with recurrent tonsillitis [11]. Numerous episodes of tonsil inflammation in a short period of time could have also been linked to possible compromised immunity, such as in the course of considered lymphoma. It might be suspected that frequent or abnormal immunological reactions to the pathogens could have taken part in the pathogenesis of KFD. There are reports of KFD coexisting with autoimmunological or hematological disorders. Therefore, we performed a wide range of diagnostic tests that excluded systemic diseases in our patient.

Because the patient’s FNAC results were normal, an excisional biopsy of a lymph node was necessary. However, Hasan *et al*. reported that, with clear symptoms and characteristic features of KFD in FNAC results, the diagnosis of KFD may be established without lymphadenectomy [12]. Yu *et al*. reported that appropriate diagnostic material can be obtained by ultrasound-guided biopsy, which can be an optimal first-line diagnostic measure [13].

Even though KFD is a self-limiting disorder with positive outcome, the most expected diagnosis with given symptoms is a hematological disease in which the prognosis may be uncertain. Watchful waiting until receiving the results of histopathological examination may be challenging for a physician treating a patient strongly suspected of having a malignancy. Introduction of aggressive treatment without the results of the microscopic examination may be harmful and is considered inappropriate. Despite alarming clinical symptoms in our patient, only symptomatic treatment had been applied until the diagnosis was made. The course of the disease in our patient was relatively mild, and therefore there was no need to introduce additional treatment (for example, steroids).

## Conclusions

In the differential diagnosis of cervical lymphadenopathy, KFD should be taken into account. It should be noted that KFD may convincingly mimic symptoms characteristic of lymphoma. The role of recurrent tonsillitis in the pathogenesis of KFD needs further investigation.

## Abbreviations

ANA: Antinuclear antibodies
aPTT: Activated partial thromboplastin time
CD: Cluster of differentiation
CRP: C-reactive protein
CT: Computed tomography
EBV: Epstein-Barr virus
ESR: Erythrocyte sedimentation rate
FNAC: Fine-needle aspiration cytology
HB: Hepatitis B
HBs: Hepatitis B surface antigen
HHV: Human herpesvirus
IgG: Immunoglobulin G
IgM: Immunoglobulin M
KFD: Kikuchi-Fujimoto disease
LDH: Lactate dehydrogenase
PLT: Platelet count
WBC: White blood cell count
